# Assessing the Role of Vocal Plasticity in Sociospatial Coordination

**DOI:** 10.3390/ani16060890

**Published:** 2026-03-12

**Authors:** Eduardo Mercado, Julia Hyland Bruno

**Affiliations:** 1 Department of Psychology, University at Buffalo, State University of New York, Buffalo, NY 14260, USA; 2 Federated Department of Biological Sciences, New Jersey Institute of Technology, Newark, NJ 07102, USA; julia.hylandbruno@njit.edu

**Keywords:** active sensing, auditory plasticity, cetacean, communication, spatial hearing, sound localization, vocal learning, vocal interaction

## Abstract

Studies of vocal communication often focus on the messages that calls and songs convey related to reproductive activities, foraging, predator avoidance, social bonding, individual recognition, and conflict resolution. We consider ways in which vocalizations may dynamically mediate social interactions at a more basic level, through collective coordination of movements and the enhancement of spatial perception. From this perspective, animals may vocalize to probe the locations, movements, and intentions of others, to manipulate position changes by listeners, or to increase their own capacity to localize sounds. An animal’s capacity to flexibly adjust vocalizations, both in real-time and over longer periods, can increase their ability to monitor and influence conspecifics independently of any information that may be encoded within those vocalizations. Beyond simply conveying messages, reproductive fitness, or emotional states, an animal’s ability to modulate vocalizations may dynamically affect its future action plans and social roles within a group. Identifying situational, life-history, and sociospatial factors that determine how animals vocally interact in real-time is key to understanding how an animal’s vocalizations relate to its own actions and the actions of others.

## Simple Summary

Some animals such as dolphins and birds show remarkable vocal plasticity, including the ability to vocally imitate sounds. Flexible vocal skills are widely thought to provide evidence of complex social communication and advanced cognitive mechanisms. Many studies of calls and songs focus on decoding the information that listeners obtain from received sounds, including the fitness and identity of vocal individuals. Less attention has been given to understanding how vocal plasticity might contribute to collective action coordination among individuals. One way that vocal learning can contribute to group dynamics is by making it easier for individuals to track others’ movements. Vocal learning, including vocal imitation, can enhance spatial hearing by increasing a vocalizer’s ability to extract signals from noise and localize sound sources. Through precise vocal adjustments and spatial coordination of sound production, group members can potentially orchestrate complex collective actions, even if none of the vocalizations encode any specific message. Understanding when and how animals vocally coordinate their actions is critical to determining the functions of vocal imitation abilities and vocal plasticity more generally.

## 1. Introduction

Vocal signaling dominates most people’s social interactions from an early age. Parents coo at their infants, instruct toddlers, patiently endure babbling commentary, and sometimes even converse with their offspring. Children yell at their peers, sing along with their classmates, and laugh at dad jokes. The ubiquitous use of vocal communication by humans has sparked a broad search for comparable processes in non-humans. The field of animal bioacoustics in particular focuses heavily on identifying various ways that different species exchange and interpret vocal signals. The underlying premise of such studies is that by providing or collecting information about the internal states of conspecifics, animals can improve their chances at surviving and reproducing. Vocal communication provides a way for individuals to collect and transmit such information in situations where visual cues are absent or insufficient.

Initially, researchers treated animals’ vocal signals somewhat like hieroglyphics—obscure representations with partially hidden meanings. The development of spectrographic techniques solidified this mindset by providing detailed glyphs that researchers could link to behavioral states. Eventually, researchers became aware that many of the features of animals’ vocalizations were determined by their environments and the social contexts within which they lived [1,2], somewhat like the beak variations in finches described by Darwin. From this perspective, variations in vocalizations could reflect the physics of sound propagation rather than (or in addition to) any informational differences they might encode. For instance, a quietly produced vocalization intended for a nearby recipient might be acoustically different from a vocalization containing the same information that is directed toward a distant listener [3,4]. Similarly, echolocating bats closing in on a target produce vocal signals that differ substantially from those they produce while searching for targets, even though both vocalizations function primarily to inform the bat about the location and movements of prey [5,6]. And identical echolocation signals may convey quite different information depending on whether a target is present or absent. Such complexities make interpreting the functional roles of animals’ vocalizations a more difficult task than simply mapping meanings onto sound types.

**Figure 1:**
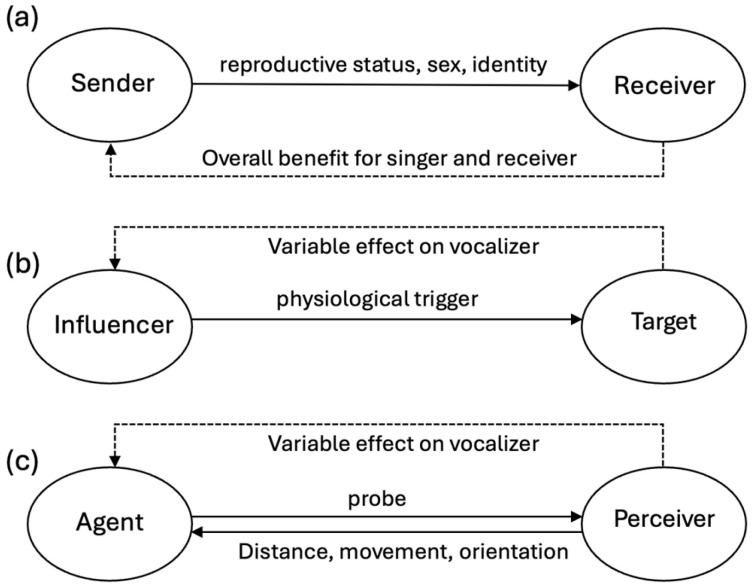
Three models of vocal interactions: (**a**) message transmission; (**b**) listener manipulation; (**c**) information seeking. The message transmission model emphasizes information encoded within sounds (signals) and how listeners use such information to make decisions. The listener manipulation model, or assessment/management framework [8], instead focuses on reactions provoked by vocalizers. When vocal interactions are construed as more exploratory, information seeking actions (e.g., analogous to echolocation), then vocalizations need not encode messages or trigger physiological reactions to mediate either decision-making or dynamic spatial coordination of collective movements.

Two conceptual frameworks dominate past and current studies of animal vocal interactions [7,8,9]. In one framework, vocalizers deliver behaviorally relevant, encoded information to listeners that enhances the sound producer’s overall reproductive success (Figure 1a). According to the second framework, vocalizing animals produce sounds not to transmit messages to listeners, but to influence others’ actions (Figure 1b).

The latter perspective focuses more on reactions evoked by sounds that require minimal signal decoding by listeners, such as when a loud scream evokes freezing and head orienting actions or when an erotic moan provokes sexual arousal. Both frameworks are often adequate for explaining vocal signaling when a single individual is producing sounds in highly specific contexts, such as when birds produce alarm calls. Interpreting the functional roles vocal signals play when multiple individuals are involved is more difficult. For instance, when many birds contribute to a dawn chorus, the types of messages being exchanged (and the individuals exchanging them) are often difficult for researchers to disentangle [10]. Even in contexts where vocal competitions seem likely, such as during territorial interactions, the specific ways that counter-singing mediates participants’ reactions are often obscure [11,12].

In principle, researchers could characterize vocal interactions using both frameworks to determine which one best predicts or explains specific scenarios for different species. In practice, however, most studies favor the message transmission model of vocal communication. One motivation that has led researchers to focus heavily on decoding animal vocalizations is the search to understand how musical and linguistic aptitudes evolved in humans. Vocal signals that seem to share structural or functional properties with spoken language (e.g., syntactical rules) or musical performances (e.g., melodic and rhythmic patterns) are viewed by some researchers as possessing greater sophistication or complexity and as requiring more advanced cognitive mechanisms [13,14]. In contrast, properties of sound production that depend less on social learning or that are thought to be reflexive are often described as simple and automatic.

Many of the communicative roles that researchers have attributed to vocalizations in past research may be incidental to their main functions. As just one prominent example, trains of echolocation clicks produced by bottlenose dolphins were initially categorized as the “rusty hinge” sound or the “creaking door” sound [15]. Rusty hinges can be perceptually sorted into subtypes that are correlated with different behavioral contexts because dolphins echolocate using different click patterns depending on the distance, size, and movements of potential targets [16,17]. These variants often reflect changes in the vocalizer’s position relative to a target of interest rather than variations in encoded messages. Such positional correlations are often impossible to detect from recordings because they relate to changing distances between the dolphin and the moving or stationary target being echolocated rather than to social interactions between dolphins [6,18]. More generally, it is difficult to identify many spatial, environmental, or context-dependent correlates of vocalizations from their acoustic properties alone. Both direct and indirect spatial cues relevant to interpreting vocal interactions are likely to be situationally dependent. Observant field researchers rarely have the knowledge and tools needed to account for all these factors, whereas the vocalizers themselves presumably have evolved many mechanisms to note and make use of all these conditional factors.

Bioacousticians have expended much more effort on characterizing the kinds of sounds and sound sequences vocalizers produce (e.g., [19]) than on identifying how animals adjust vocal production to account for situational contexts. Knowing when and where animals vocalize can, however, be critical to understanding why they vocalize and how they select which acoustic signals to produce. These contexts include the various soundscapes within which they vocalize, including vocalizations they recently have produced and heard. For example, vocal repetition sometimes results from a persistent trigger like the presence of a predator. But in other cases, repetition functions to provide dynamic cues about a vocalizer’s movements, or, in the case of echolocating bats, dynamic cues about the locations and actions of potential prey. Sometimes, repetition reflects simple ontogenetic constraints, as in the reduplicated babbling of infants and young songbirds that gets replaced by variegated vocal sequences as learners slowly acquire the transitions between sounds in their repertoires [20]. The capacity for context-dependent vocal control and coordination may also change during ontogeny, as a result of a vocalizer’s experiences interacting with specific soundscapes as well as under the influence of other physical, motor, and social developmental processes.

Here, we argue that animal vocalizations allow individuals to sense, track, and influence each other’s behavior and movements in real time, actively shaping and coordinating social actions and interactions, regardless of whether the vocal signals encode overt messages or emotional states (Figure 1c). In the proposed framework, vocalizations may serve as adjustable tools that individuals can use to co-construct complex action sequences (see also [21,22]). From this perspective, both agents (vocalizers) and perceivers (listeners) interact in dynamic, spatially contingent ways using mechanisms that parallel processes typically associated with echolocation (i.e., by continuously monitoring and vocally responding to spatial and situational variations in real-time). We first provide examples in which animals vocalize while rapidly coordinating directional movements as a group. Next, we describe how vocal plasticity can potentially enhance animals’ abilities to vocally regulate coordinated actions by refining their spatial hearing. Finally, we illustrate how this framework can be applied to a particularly flexible vocalizer, the humpback whale.

## 2. Vocal Flexibility and Movement Coordination

Interest in the role vocalizations play in regulating social interactions has increased dramatically over the last three decades [23]. Vocal signals are associated with the initiation of flight by groups of birds [24], cooperative foraging [25], maintenance of contact [26], and collective actions [27]. A recent study of flocks of zebra finches flying together in a wind tunnel revealed that individuals intermittently used in-flight vocalizations to coordinate flight patterns and avoid collisions [28]. Birds in flight need to make rapid decisions about adjustments to flight trajectories in three dimensions. Zebra finches rarely called while in flight within the wind tunnel. But when an individual did call, it did so just before flying upward. A bird was most likely to produce a “stack call” when its initial position was lower within a flock, suggesting that individuals may have vocalized to indicate to other birds in the flock where and when it was going to move (i.e., sending a message warning others of an impending movement). Alternatively, the stack call might be a reaction to nervousness that indirectly triggers surrounding birds to fly higher, or a probe that the caller uses to seek information regarding when moving up is an option. Flocking birds typically rely on vision to coordinate flight movements. They cannot, however, visually attend panoramically in all directions simultaneously. Vocal signals provide spatial cues that may enable multiple listeners to collectively coordinate changes in position and to maintain stable spacing while in flight [28]. Importantly, zebra finch stack calls were only interpretable as possible flight directives when researchers observed where the caller and non-calling flock mates were when the calls were being produced. More controlled experiments would be needed to determine whether callers are voluntarily signaling their desired future spatial relocations, reacting to increased arousal, or attempting to assess the locations of other nearby birds.

**Figure 2:**
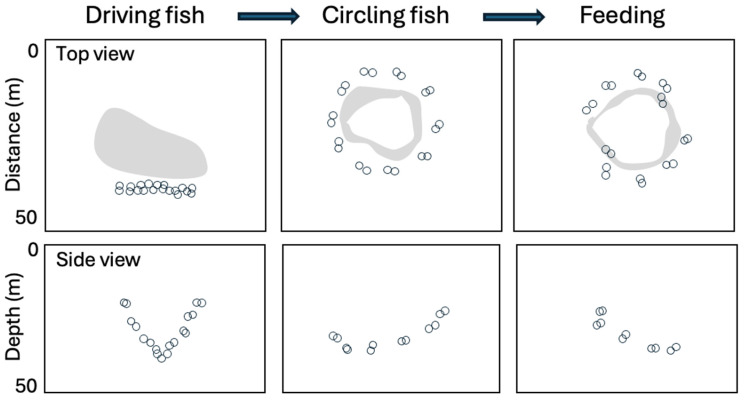
Spinner dolphins forage at night in groups following distinct phases of coordinated actions. Multiple pairs of dolphins initially swim together in a line and undulate as a group moving up and down a few meters, as well as forward, gradually increasing the density of fish ahead of the line (leftmost panels; circles denote individual dolphins and gray shading corresponds to fish). Next, dolphin pairs rapidly encircle the fish (middle panels). Finally, two pairs of dolphins on opposite sides of the circle move into the circle at the same time to catch fish (for about 10 s) before returning to their positions in the rotating pattern (rightmost panels), after which the two pairs swimming behind the most recent feeders enter the circle. High rates of clicking occur when the pairs begin driving fish and when they switch to circling the fish (Data from [34]).

Most current discussions of vocal regulation of group dynamics assume that senders vocally encode messages for listeners to use to make decisions (e.g., [27,29]). These messages could include information about species, sex, individuality, emotional states, and the presence of prey or predators. Preconceptions about the kinds of information vocal signals are designed to encode do not, however, always accurately predict vocal interactions between group members. For instance, dolphins are widely thought to engage in sophisticated social vocal interactions in which they exchange information about their identities, emotional states, locations, and desire for contact [30,31,32]. Consequently, one might expect that when dolphins coordinate their foraging efforts in contexts where vision is limited, they might rely heavily on vocal interactions to achieve success. For this reason, when researchers began observing groups of spinner dolphins synchronizing their movements as the dolphins cooperatively herded fish at night (Figure 2), they suspected the dolphins might use whistle-based communication to coordinate their actions [33,34]. Acoustic observations of foraging bouts revealed, however, a notable absence of any whistles. Even more surprisingly, the spinner dolphins appeared to produce relatively few echolocation clicks when actively capturing fish. Instead, the dolphins produced clicks mainly when transitioning between stages of corralling the fish, specifically when they shifted, as a group, their swimming patterns and positions (Figure 2). It is not known how producing clicks during shifts in strategies might contribute to successful cooperative foraging or if all or only a subset of the dolphins are clicking during transitions.

Pairs of dolphins within the group were spaced such that visual monitoring of other group members’ positions was likely not an option. Given that the dolphins’ movements were coordinated in three dimensions, tracking of other animals’ positions in real-time almost certainly played a role. Click production could produce echoes revealing the changing locations of group members as well as the movements of the click producers and surrounding prey. Cues from clicks might thus provide spatial information to foraging spinner dolphins analogous to what most mammals access visually (i.e., the clicks might be used to construct a perceptual scene that guides future actions, rather than to transmit messages). Other modalities might also play a role in formation initiation or maintenance. For example, a dolphin might feel wakes produced by other dolphins’ that could reveal their recent trajectories.

Dolphins are highly versatile vocalizers. Spinner dolphins in particular produce a large repertoire of vocalizations in many different contexts [35,36], including situations where individuals can visually monitor other group members. Why, then, are these dolphins abandoning whistle-based communication in a situation where their movements need to be tightly coordinated in three dimensions over tens of meters in the dark? One possibility is that most of the information hypothesized to be encoded in dolphin whistles is superfluous in this foraging context. Individuals are likely familiar with who is in the group, what sex and species they are, what their emotional and motivational state is likely to be, and the fact that prey is currently present. Rather than spending time whistling to “state the obvious,” foraging spinner dolphins may be collectively seeking information about contextual changes or for consensus about when to shift strategies or positions. In this context, clicks might serve a similar role to stack calls in flocking zebra finches, acting as trajectorial directives that enable group members to shift their movements in concert. The examples of zebra finch stack calls and spinner dolphin clicks during fish herding illustrate that vocalizations can contribute to movement coordination in complex ways not accounted for by current models of animal communication.

Bioacousticians traditionally have considered clicks to be limited as message-bearing signals because of their acoustic simplicity. But what clicks lack in complexity they more than make up for in their potential for revealing high-resolution spatial information (as evidenced by their use in echolocation) when and if listeners have neural circuits capable of extracting that information. Collecting measures of detailed neural responses in spinner dolphins is currently infeasible for multiple reasons. Behavioral studies of their close relatives, however, have definitively established dolphins’ impressive capacities for auditory spatial localization [18,37]. As with other echolocating animals, dolphins’ hearing capacities match their productive versatility. Unlike other echolocating animals, dolphins and other cetaceans also possess exceptional vocal learning skills [38]. Past theories for why this is so have focused on the additional opportunities for flexible information encoding that vocal learning provides [30]. The possibility that vocal learning might also (or alternatively) increase cetaceans’ ability to extract spatial information from sounds has largely been overlooked. More generally, few researchers have considered how vocal plasticity might contribute to animals’ coordination of dynamic actions. In the following section, we review various ways that vocal flexibility can alter listeners’ abilities to track, direct, and anticipate the movements of vocalizers within a group.

## 3. Adjusting Vocalizations to Enhance Spatial Hearing

**Figure 3:**
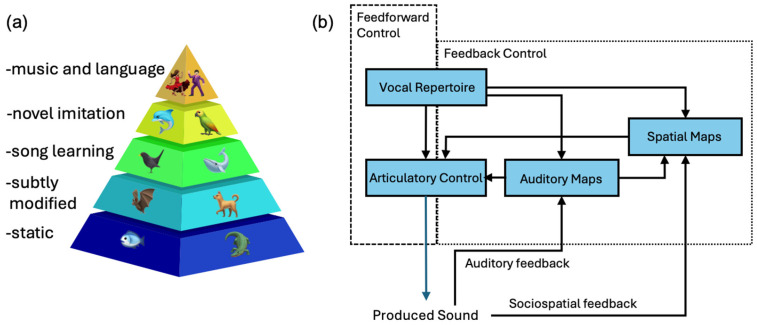
Conceptualizations of vocal plasticity. (**a**) A hierarchical vocal learning taxonomy based on sound production mechanisms in which humans are at the apex of cognitive control of vocal flexibility (adapted from [14]). (**b**) A control systems model of vocal production in which both imitation and vocal adjustments are mediated by multiple, dynamically interacting subsystems (adapted from [39]). In this model, an individual’s vocal repertoire affects the precision with which complex sounds can be spatially localized.

Recent discussions of the relationship between vocal plasticity and social communication emphasize evolutionary advancements in voluntary motor control, specifically the emergence of mechanisms for flexibly stretching vibrating membranes and for shaping resonating cavities [14]. Differences in the time frames over which animals adjust their vocal sounds and sequences have been interpreted as markers of vocal complexity and flexibility [13,14]. Tyack [13], for instance, dichotomizes vocal learning processes as being either limited or complex based on whether the learning involves comparing perceived sounds to long-lasting auditory templates learned from models; vocal adjustments that involve matching sounds to learned templates are classified as complex. Vocal matching may involve producing similar sounds and/or similar sequences of learned or species-typical sounds. Instances in which an animal modifies sounds based on current or recent experiences, such as the adjustments made by echolocating bats, are considered to be less complex (limited) because no long-lasting, memorized auditory templates are being used to guide vocal production. Another popular taxonomy of vocal learning distinguishes “subtle modifications” to vocalizations from vocal imitation of novel sounds [14]. This continuum of vocal adjustments is characterized as mapping directly onto levels of cognitive demands (Figure 3a), such that subtle changes to vocalizations require little cognitive capacity while vocal imitation may rely on extensive cognitive sophistication.

Vocal learning taxonomies emphasize variations in observed performances and hypothesized cognitive capacities across different species [40,41,42,43,44]. The main goal of such taxonomies is to facilitate cross-species comparisons that will elucidate the evolutionary histories of vocal capabilities in vertebrates. Of the many vocal learning variants, far more is known about the neural mechanism of imitative song learning in songbirds than other behavioral processes or other taxa, and in the neuroscience of birdsong only a handful of the ~4000 songbird species have been studied [45]. Control systems models (Figure 3b) provide a more mechanistic approach to characterizing vocal plasticity [46,47]. In this model, an individual’s ability to control vocal production and to imitate sounds gradually develops through experience and practice. Such models highlight the many interacting processes that can affect the flexibility with which individuals produce *and perceive* sounds while remaining neutral with respect to the relative cognitive sophistication required to control vocal adjustments in the short- or long-term.

The relative strengths and weaknesses of these two approaches to conceptualizing vocal plasticity are particularly evident in the case of bats. Echolocating bats are some of the most flexible vocalizers in the animal kingdom. And yet, according to the vocal learning taxonomy shown in Figure 3a, their vocal skills are less sophisticated than singing by songbirds or whistle production by dolphins. The rapid rates at which echolocating bats adjust the form and timing of their vocalizations gives a sense of automaticity that obscures just how vocally versatile bats are. No one has tested how flexibly bats raised in isolated chambers can echolocate. Consequently, the role learning plays in bats’ vocal development remains unknown. Some bats produce songs of comparable structural complexity to those sung by birds [48,49] and adjust properties of their calls to match sounds they hear being produced by conspecifics [50,51,52,53,54,55]. Any learning mechanisms that might contribute to flexible bat echolocation (see, e.g., [6,56]) typically are attributed to more primitive vocal motor control systems [57].

Bat echolocation involves vocal control beyond that seen in dolphins because bats continuously adjust multiple acoustic features of their calls as a function of their position relative to flying insects and obstacles in their environment [6,56,58]. Accounting for the dynamic interactions between bats’ outgoing calls and incoming spatial updates is critical to understanding the various ways that different bat species modulate vocalizations. Songbirds may similarly modulate vocalizations in response to ongoing sociospatial dynamics [59,60] in ways that may depend on vocal learning [61].

Vocal learning has been studied independently of action coordination under the assumption that a vocalizer’s movements serve mainly to put an individual in a location where messages can be effectively transmitted to behaviorally relevant recipients [62,63]. The ability of animals to spatially localize vocalizers and judge their positions and movements in three dimensions typically is taken as a given. Precisely judging a vocalizer’s distance and elevation is perceptually non-trivial, however, and may depend on auditory learning and plasticity [64,65].

The extent to which different species learn to localize the calls and songs within their vocal repertoire is essentially unknown. Morton [66] proposed that spatial hearing abilities could be a major evolutionary driver of song learning by birds. Certainly, recognizing whether a singer has crossed a territorial boundary requires the capacity to judge auditory distance relatively precisely, a capacity which in humans depends on a listener’s familiarity with the to-be-localized sound [67]. Similar sound localization needs associated with action coordination in cetaceans may also explain their convergent evolution of flexible vocal learning mechanisms [38].

The ability to precisely track movements of a sound source, both in terms of absolute locations and relative to the position of a listener, depends on familiarity with the details of received sounds [68,69,70]. For animals that rapidly and routinely coordinate their movements in three dimensions, exquisite spatial hearing is fundamental for the vocal regulation of collective dynamics [23,27]. How might vocal plasticity contribute to spatial hearing in ways that facilitate sociospatial coordination? Consider the vocal learning skills of zebra finches. The songs of zebra finches show complex variations across individual singers, variations which are known to be acquired through experience during development [71]. These complex features of zebra finch songs are thought to provide male singers with distinctive acoustic badges to which female listeners may show idiosyncratic preferences [72]. Consequently, zebra finch songs are often described as fixed acoustic displays (i.e., messages sent to indicate reproductive fitness) that females subjectively judge when comparing males [45]. Males lock in on a stereotyped song form relatively early in development, leading researchers to describe zebra finches as closed-ended learners.

Males do not limit song production to situations in which a female is present, however [73]. Songs produced in the absence of females are classified as undirected songs [74]. Undirected songs are often interpreted as practice for later serenades directed toward females. This portrayal of zebra finch singing assumes that males will put their best beak forward and display their peak vocal skills in the presence of females, much like peacocks display their tail feathers. Contrary to this characterization, singing males appear to modify their singing based on the vocal actions of listening females [45]. A singing zebra finch will also modify the timing and features of song bout production after spending time with another adult male, in ways that increase the similarity of song bout production across the two males [75]. It thus appears that zebra finches dynamically adjust some properties of learned songs and how they use those songs in ways that accommodate the specific socio-acoustic circumstances they encounter. Although the functional goals of such partner-based vocal accommodation are unclear, the fact that “closed-ended” vocal learners continue modulating song production as adults provides clear evidence that flexible vocal production skills remain relevant throughout a zebra finch’s life and may point to previously unsuspected functions of coordinated vocalizations and vocal learning in zebra finches and other songbirds. Zebra finches sing continuously in contexts where multiple mated pairs move together as a group [76,77], suggesting that the capacity to monitor the changing positions of familiar vocalizing conspecifics may facilitate movement coordination.

Consider also the vocal learning skills of orcas. Decades of recordings of orcas vocalizing in different locales revealed that local pods of orcas each show distinctive vocal dialects [78]. Because variations in the pods’ vocal repertoires are not predicted by geographical separation, genetics, or environmental conditions, most researchers assume that the observed dialects are the result of iterative vocal learning, particularly vocal imitation. Consistent with this possibility, wild orcas have been observed producing sounds with properties matching those of other species [79]. Similarly, a trained orca proved to be capable of copying novel sounds produced by a trainer [80]. The advantages that orca pods gain from vocal learning and use of a shared repertoire are poorly understood but generally presumed to relate to social cohesion and recognition of group members. A subset of orca calls are known to contain acoustic features that facilitate monitoring of the caller’s orientation and direction of movement by distant listeners [81], indicating that group members may use calls to coordinate group movements [82]. The spatial informativeness of orca calls has not previously been linked to local dialects. However, studies of auditory distance estimation in human listeners show that spatial resolution of familiar learned sounds exceeds that of time-reversed versions of those same sounds [83], suggesting that use of a shared group-specific vocal repertoire could potentially enhance audiospatial tracking in listeners familiar with the repertoire while simultaneously obfuscating source localization by listeners unfamiliar with that vocal repertoire.

Past studies of orca calls have focused on cataloguing variations in vocal repertoires produced by different groups, on documenting the relative stability of locally shared repertoires over time [82], and on using calls to distinguish between orca ecotypes [84,85,86,87]. It is widely assumed that call repertoire differences between groups serve mainly to communicate information about group identity [88]. Orca calls are frequently described as stereotyped, suggesting that any vocal adjustments that individuals make in different situations are either subtle or not salient to listening humans. In this respect, orca calls may be more similar to the clicks used by foraging spinner dolphins than to bat sonar calls or zebra finch songs. Detailed acoustic analyses of orca calls do, however, show variations in frequency content, duration, and frequency modulation across calls of the same categorical type [89]. Whether such variations are actively controlled by orcas or affect the functionality or spatial localizability of individual calls is unknown. The degree to which familiarity with a specific call determines an individual group member’s ability to spatially localize a vocalizer is also unknown.

It is clear, nevertheless, that collective movements in low-visibility environments would be facilitated by the ability to perceive or anticipate the changing positions of group members. Vocal learning capacities in bats, zebra finches, and orcas suggest that flexible vocal control may enhance three-dimensional spatial monitoring when visual cues are limited. Spatial hearing undoubtedly contributes to vocal regulation of collective dynamics in such scenarios. Thus, to the extent that vocal learning can enhance auditory spatial localization and tracking of sound sources, it is likely to shape the vocal repertoires of species that rely heavily on vocalizations to guide their collective actions. This holds true not just for animals that move in cohesive, stable social groups like orcas and zebra finches, but also for species whose interactions are more spatially dispersed like baleen whales. Section 4 considers more closely how vocal learning can contribute to spatial hearing and sociospatial interactions in situations where individuals are not attempting to choreograph joint actions but are instead using vocalizations to mediate social interactions from long distances.

## 4. Vocal Regulation of Sociospatial Interactions

As noted earlier, vocal convergence within shorter-duration time windows has generally been viewed as requiring less neural and cognitive sophistication than vocal adjustments made over days, weeks, or months (such as is often observed with vocal imitation). One species often offered to illustrate this more sophisticated form of vocal learning is the humpback whale. Singing humpback whales in a particular locale converge on a predictably structured song while varying the properties of songs over time [90]. This dynamic convergence is generally interpreted as a case of cultural transmission that enables singing males to advertise their sexual prowess [91]. An alternative explanation for why singing humpback whales collectively converge on song features is that they are systematically adjusting songs in reaction to shared acoustic conditions [92,93]. This process of joint action might be similar to the coordinated shifts in flight patterns observed in large groups of starlings, if one assumes a much slower rate of song adjustment that accumulates over time [94]. Parallel adjustments to vocalizations may give the appearance of communal convergence in situations where vocalizers are neither vocally imitating sounds nor actively converging on features of vocalizations that they have heard.

Singing humpback whales that can hear other singers in the vicinity appear to modulate the acoustic properties of their songs interactively [95,96]. Singers generally do not form social groups that last more than a few days [97], which is quite different from the social contexts experienced by spinner dolphins, zebra finches, or orcas. Humpback whales’ population-wide coordination of song features across multiple time scales suggests that emergent similarities in song structure and acoustics are not serving to coordinate synchronized swimming patterns, accommodate vocal actions of familiar conspecifics, or announce membership in a particular group. Nevertheless, the flexibility with which singing humpback whales modulate song features [98] may ultimately serve to guide the coordinated spacing and relocation decisions of singers, non-singers, and larger groups of humpback whales.

Studies of songbirds show that learned vocal sequences may attract or repel listeners [99]. Such reactive relocations are generally interpreted as evidence that listeners are judging qualities of those sequences relative to some internal standard of preference or adequacy. Songbirds may also use songs, however, to flexibly coordinate their movements in real-time. For example, when singing chickadees’ movements were tracked during times when they maintained territories [12], researchers discovered that the singers were moving (covering an average distance of ~20 m between songs) in 97% of singing contests. About half the time, singers were moving away from each other while exchanging songs, suggesting that they were not actively contesting a territorial boundary. Why would familiar neighbors with established mates counter-sing as they move away from each other and away from territory boundaries? Repeatedly broadcasting songs while moving provides both singers with cues to the changing positions and movement trajectories of their neighbor, thus enabling each to monitor the others’ actions in the absence of visual cues. Auditory spatial cues may depend on the relative heights of sources and receivers, the type and density of vegetation, weather conditions, and the direction the singers are facing [1,100]. The informativeness of these cues thus varies with the habitat within which songs are produced, such that the ability of chickadees to track the movements of distant singers likely depends on their experience with hearing the same song produced from many different locations. The precision with which singers can reproduce songs may also affect how accurately songs can be localized [101,102,103].

Like singing chickadees, humpback whales are thought to use similar songs to enable listeners to make judgments about individuals’ adaptive fitness [104]. Songs may also serve to space singers [105] or to attract other whales [106]. Such functions are consistent with the possibility that singing humpbacks modulate song features to coordinate the movements of other whales, although many other species achieve similar functions using less dynamic vocal signals. It is unclear whether the spatial cues listening whales may extract from songs after long-distance transmission are primary or secondary to song function. In general, use of spatial information from vocalizations to guide one’s actions could happen independently of the functions of those vocalizations (e.g., when predators eavesdrop to find prey or prey eavesdrop to avoid predators). Songs may provide indications of a singing whale’s movements beyond just giving away their position. For instance, fin whale songs vary as a function of how rapidly the singer is swimming [107], irregularities in humpback whale songs are correlated with variations in surfacing patterns [108], and patterns within pygmy blue whale songs are predictive of the singer’s depth below the surface ([109]; see also [58]). The ability of listeners to extract and interpret such spatial cues depends partly on the consistency with which vocalizers produce sounds, especially in oceanic environments. Why then are singing humpback whales varying songs over time?

As in echolocating bats, real-time adjustments to produced sounds may increase a singing humpback whale’s ability to selectively monitor the effects of its vocalizations and/or to account for the specific constraints of the physical and social environment within which it is vocalizing. Assuming singers have some awareness of what other whales within hearing range are doing, they may also adjust song properties to spatially direct their songs towards specific individuals [110]. Given the long distances over which songs omnidirectionally propagate [111], addressing songs to individuals at prescribed locations may require greater vocal control than calling orcas need to coordinate group movements or than chickadees require to influence neighbors’ actions. The longer-term changes observed in humpback whale songs within and across years parallel acoustic changes observed in individual songs [94,112], suggesting that these changes might be an incidental effect of accumulated short-term adjustments to songs and/or a product of vocal learning processes.

## 5. Conclusions

Studies of vocal communication in animals often focus on the roles sounds can play in conveying encoded messages related to courtship, territorial defense, foraging, predator avoidance, social bonding, individual recognition, and conflict resolution. Humans clearly do more with their voices than posture, pose, and broadcast their identities. Historically, interest in humans’ unique abilities (especially those related to speech and language) has encouraged researchers to favor sender–receiver models of vocal communication. As a result, cross-species comparisons of vocal behavior often emphasize the importance of vocal flexibility for mediating complex social dynamics [14], specifically by providing animals with mechanisms for learning, encoding, and transmitting complex messages.

Commonalities in vocal learning across humans and other species have been highlighted as clear examples of overlap in sound usage [113]. Homologous neural [114] and even genetic mechanisms [115] provide support for the idea that at least some vocal learning processes are functionally comparable across taxa and reflect similar evolutionary trajectories. Evidence of vocal dialects [116], vocal imitation [117], cultural transmission [118], and coordinated sound production between conspecifics [96] provide further support for the idea that animals other than humans are learning vocal skills in ways that parallel human learning and for similar functional purposes, including potentially the intentional transmission of information. Even so, the specific scenarios that drove the evolution of vocal flexibility in some birds, marine mammals, and primates versus vocal stereotypy in most other birds and mammals are questionable. One extensively cited ultimate cause for the emergence of vocal flexibility and sophisticated vocal learning mechanisms is sexual selection [14]. Darwin [119] specifically suggested sexual competitions as the spark that led to the vocal prowess of humans: “musical notes and rhythm were first acquired by the male or female progenitors of mankind for the sake of charming the opposite sex … We may go even further than this, and … believe that musical sounds afforded one of the bases for the development of language.” (p. 737) (see also [120]). The limitations of sexual selection as an explanation for humans’ vocal skills have been widely noted [121,122]. In contrast, few authors have questioned the adequacy of this proposed evolutionary force in explaining vocal flexibility in non-primates.

Vocal learning mechanisms can support language learning, enhance problem-solving abilities, and ultimately enable the emergence of cultures. In this way, vocal learning may facilitate message transmission, allowing for more cognitively complex exchanges. The main evidence used to support claimed links between vocal sophistication and vocal learning, however, is the observation that only humans and a few other animals show the ability to use stored auditory templates to guide their vocal production [13]. There is evidence from studies of jazz musicians showing that cortical networks are also engaged during improvisational sound production [123], which is less clearly related to matching stored auditory templates. The kinds of rapid, situation-dependent adjustments made by performing improvisors coordinating rhythms and melodies are more analogous to the kinds of flexible adjustments made by echolocating bats than to the typically stereotyped learned songs produced by vocally learning birds. Certainly, the coordinated sequences produced by jazz musicians are at least as sophisticated as any sound sequences produced by singing birds or whales, regardless of their conformity to any learned templates (or any dependence on vocal learning per se). Such musical coordination requires precisely timed finger movements guided by auditory impressions and extensive experience with the likely actions of ensemble members. Similarly, vocalizations that birds use to coordinate their flight movements in flocks in the absence of vision may require precise monitoring of who is going where at any given moment. In both cases, coordination is multimodal and depends in part on flexible sound-producing skills and acquired social knowledge.

Less attention has been given to evolutionary advances in auditory perception and memory that might render changes in vocal production circuits advantageous, despite studies showing that developing infants recognize words, rhythms, and even dialects prior to showing any evidence of flexible vocal production capacities [124]. The fact that auditory processing abilities evolved prior to any vocal signaling capacities is well established [125] and easy to understand; vibrations provide information that can promote survival. It is less obvious how hearing might catalyze variations in vocal flexibility or vocal imitation abilities, especially given that most vertebrates hear, while only a few imitate sounds. Sexual selection of vocal displays requires the ability to hear and recognize distinctions between potential mates or competitors, but this does not necessitate any specialized auditory abilities. In contrast, animals’ use of echoes to recognize and track objects clearly demands spatial hearing powers that go beyond what most hearing species can manage. The ultimate evolutionary reason that bats constantly adjust their signals while echolocating is because they can interpret subtle variations in the streams of echoes they receive as behaviorally relevant events happen around them. The essence of echolocation is perceptual. For bats, spatial hearing needs are the primary drivers of vocal flexibility. Adaptations related to spatial hearing might also have facilitated the evolution of vocal imitation abilities in cetaceans, birds, and humans [38].

Understanding how variations in spatial hearing abilities might ground both vocal convergence and vocal imitation abilities requires a close examination of the environmental and social contexts within which animals are adjusting and copying vocalizations. Identifying situational similarities during vocal interactions is key to determining when vocal changes serve to: (a) inform conspecifics of the vocalizer’s status/state; (b) manipulate the actions of listeners; or (c) provide peers with a spatial hearing aid that facilitates the coordination of joint movements. Many vocal signals currently assumed to encode messages may instead (or additionally) mediate social interactions at a more basic level [22], through coordination of movements and actions via spatial perception. Animals may vocalize to supplement visual indications of position changes; to probe the locations, movements, and intentions of others; or to attempt to change others’ actions. In this way, vocalizations may dynamically affect a vocalizer’s action plans and social roles. Just as echolocating bats will vary their search signals dynamically based on their altitude [58] and the positions of nearby obstacles [5], other vocalizing animals may select when, where, and how to vocalize depending on their goals and the perceived actions of others around them. Echolocating species commonly modulate the timing of sound production relative to their own movements and in some cases may control their trajectories based on the vocalizations of conspecifics [6]. Singing birds and whales may use similar strategies in their vocal interactions. Characterizing situational and sociospatial factors that determine how animals vocally interact in real-time is necessary for revealing how animals’ vocalizations guide self-selection of actions and influence the actions of others.

In summary, past emphasis on the message-carrying functions of animal vocalizations has led researchers to overlook other ways that vocalizations may regulate group dynamics. Some vocalizations currently assumed to function primarily as contact calls, sexual advertisements, or emotional expressions may instead (or additionally) serve as markers of movements creating new possibilities for dynamic action coordination. In this context, vocal plasticity can potentially enhance the control of collective movements.

## Data Availability

No new data were created.
